# Factors affecting the outcome of hospitalization among liver cirrhosis patients

**DOI:** 10.12669/pjms.35.5.884

**Published:** 2019

**Authors:** Muhammad Irfan, Yasir Mahmud, Rana Muhammad Suhail Khan, Qamar Rafiq, Muhammad Arif Nadeem, Aftab Mohsin

**Affiliations:** 1 Dr. Muhammad Irfan, FCPS (Medicine), FCPS (Gastroenterology). Associate Professor of Medicine, Gujranwala Medical College, Teaching Hospital, Gujranwala, Pakistan; 2 Dr. Yasir Mahmud, FCPS (Medicine). Senior Registrar, Services Institute of Medical Sciences, Services Hospital, Lahore, Pakistan; 3 Dr. Rana Muhammad Suhail Khan, FCPS (Medicine). Senior Registrar, Allama Iqbal Medical College, Jinnah Hospital, Lahore, Pakistan; 4 Prof. Dr. Qamar Rafiqa, FCPS (Medicine). Assistant Professor, Gujranwala Medical College, Teaching Hospital, Gujranwala, Pakistan; 5 Prof. Dr. Muhammad Arif Nadeem, FCPS (Medicine). Professor of Medicine & Gastroenterology, Services Institute of Medical Sciences, Services Hospital, Lahore, Pakistan; 6 Prof. Dr. Aftab Mohsin, FRCP. Professor of Medicine, Allama Iqbal Medical College, Jinnah Hospital, Lahore, Pakistan

**Keywords:** Liver cirrhosis, Outcome of hospitalization, Pulmonary aspiration, Age groups, SPSS

## Abstract

**Objectives:**

To determine the factors affecting the outcome of hospitalization in patients suffering liver cirrhosis hospitalized to tertiary care hospital, Gujranwala, Pakistan.

**Methods:**

After informed consent, the data of liver cirrhosis patients with age >12 years hospitalized from June 2016 to May 2017 was collected by purposive sampling. The outcome of the hospitalization in term of ‘death’ and ‘no death’ was noted. Statistical analysis was done using SPSS version 25. Bivariate analysis as well binary logistic regression was performed to ascertain the effect of different predictors like gender, age, history of diabetes mellitus, etiology of cirrhosis, presence of hepatic encephalopathy at presentation, presence of upper GI bleed, and tracheobronchial aspiration on the likelihood that death would be the outcome in liver cirrhosis patients.

**Results:**

Amongst total of 1304 patients, 15.7% died during hospitalization. The mean age of those who died was 58.08 + 14.49 years. Bivariate analysis suggested that mortality was significantly higher in group of patients who had hepatic encephalopathy at presentation (p<0.01), no upper gi bleed (p<0.01), and who got tracheobronchial aspiration during hospitalization (p<0.01). It did not differ significantly in male/female gender (p=0.504), diabetic/non-diabetic groups (p=0.652), with viral/non-viral etiology of cirrhosis (p=0.918). Binary logistic regression revealed that patients who had tracheobronchial aspiration were 12.392 times more likely to die than who had no tracheobronchial aspiration. Similarly, patients who presented in hepatic encephalopathy were 7.862 times more likely to die than who presented without hepatic encephalopathy.

**Conclusion:**

The inpatient mortality rate amongst cirrhotic patients was high. Age, gender, history of diabetes, viral etiology of cirrhosis did not significantly contribute in the mortality of these patients. The patients who presented in hepatic encephalopathy, and who suffered tracheobronchial aspiration during hospitalization were more likely to die. Excellence in hepatic encephalopathy management and prevention from aspiration can effectively reduce the mortality rate of cirrhotic patients in our hospitals.

## INTRODUCTION

Liver cirrhosis is an important public health issue in Pakistan.^1^ The HCV infections is the commonest etiology of liver cirrhosis in our country.^2^ Its world-wide prevalence ranges from 4.5% to 9.5%.^3^ The reasons for hospitalization of cirrhotic patients include multiple complications of this disease, e.g. upper gastrointestinal bleed (UGIB)^4^, hepatic encephalopathy (HE)^5^ etc. The outcome of the hospitalization of these patients in term of recovery or death is unsatisfactory. In-hospital mortality among patients suffering liver cirrhosis is high world-wide, ranging from 13.5% to 35%.^6^,^7^ Previously, different prognostic models^8^ like the Child-Turcotte-Pugh (CTP) scoring system and the model for end-stage liver disease (MELD) had been formulated for end stage liver disease. However, till now, patient and hospital related factors affecting death rate or in-hospital mortality in admitted liver cirrhosis patients are poorly understood.

If some etiological factors are found in our set-ups, then preventive measures would reduce the death among hospitalized cirrhotic and thus outcome of hospitalization could be improved. This will, in turn, lend support to clinical and healthcare decision-making, as well as to the creation and adaptation of policies based on the facts. Therefore, the aim of the present study was to determine the factors affecting the outcome of hospitalization in patients suffering liver cirrhosis hospitalized to tertiary care hospital, Gujranwala, Pakistan.

## METHODS

This cross-sectional study^9^ was conducted in the Department of Medicine Unit 1, GMC Teaching Hospital, Gujranwala from June 2016 to May 2017. After approval from the ethical review committee (ERC) of the institution, written informed consent was taken from all patients. Sample size calculation was performed using online Rao soft calculator. With a population size of 20000, response distribution of 50% and confidence interval of 95%, the minimum recommended sample size was 377. The data was collected prospectively by purposive sampling using a structured proforma. All the diagnosed CLD patients with age greater than 12 years who were hospitalized for different complications of liver cirrhosis were included in this study. The outcome of the hospitalization of all the patients in term of ‘death’ and ‘no death’ was also noted. Second group included the patients who got discharge, referred to other institutes or leaved against medical advised. Pulmonary aspiration^10^ was labelled after finding tachypnea, wheeze, crackles on chest examination, recovering the contents like food particles during direct suction of the airways, with or without finding opacities on x-ray chest posteroanterior view.

Statistical analysis was performed using the Statistical Package for Social Science (SPSS), version 25. Age of the patients was the only quantitative variable, while gender, history of diabetes mellitus, etiology of cirrhosis in term of viral or non-viral, presence of upper GI bleed, hepatic encephalopathy at presentation, and tracheobronchial aspiration were the qualitative variables. During descriptive interpretation of data, continuous variables were expressed as mean and standard deviation. Frequencies and percentages were computed for different categorical variables. Independent sample T test was used to compare the mean age of patients in two outcome groups (death/ no death). Bivariate analysis was performed to find the predictors of mortality amongst liver cirrhosis patients using the chi-square test of independence. All p-values were two sided and considered as statistically significant if < 0.05. Odds ratios and confidence interval were also calculated. The binary logistic regression was also performed to ascertain the effect of different predictors on the likelihood that death would be the outcome of the hospitalization in liver cirrhosis patients.

## RESULTS

Amongst 1304 liver cirrhosis patients, 15.7% (n=205) died while 84.3% (n=1099) not died during hospitalization. The mean age of the patients who died was 58.08 ± 14.49 years and the mean age of the patients who not died during hospitalization was 53.85 ± 14.19 years. The mean difference of the outcome of the hospitalization patient who was died and who survives due to liver cirrhosis is 4.23 and the comparison result is statistically significant (p<0.01) (Table-I).

**Table I:** Comparison of mean age of patients suffering liver cirrhosis with outcome of hospitalization (death/no death) (n =1304)*.

Outcome of hospitalization	Mean Age (Years)	Standard deviation	Mean difference	p-value	95% Confidence interval
*Death*	*58.08*	*14.491*	*4.238*	*<0.01*	*2.113 – 6.363*
*No Death*	*53.85*	*14.191*			

* Independent sample T-test was used

Bivariate analysis suggested that mortality was significantly higher in group of patients who had hepatic encephalopathy at presentation (p<0.01), no upper GI bleed (p<0.01), and who got tracheobronchial aspiration during hospitalization (p<0.01). It did not differ significantly in male/female gender (p=0.504), diabetic/non-diabetic groups (p=0.652), with viral/non-viral etiology of cirrhosis (p=0.918) (Table-II).

**Table II:** Factors affecting the outcome of hospitalization in patients suffering liver cirrhosis (n = 1304)*.

Factors	Outcome of hospitalization		Total	p-value

	Death	No death		
***Gender:***				
Male	99 (48.3%)	528(48.0%)	627 (48.1%)	0.504
Female	106 (51.7%)	571(52.0%)	677 (51.9%)	
***Diabetes Mellitus:***				
Yes	29 (14.1%)	142(12.9%)	171 (13.1%)	0.652
No	176 (85.9%)	957(87.1%)	1133(86.9%)	
***Etiology of cirrhosis***				
Viral	173 (84.4%)	922(83.9%)	1095(84.0%)	0.918
No-viral	32 (15.6%)	177(16.1%)	209 (16.0%)	
***Hepatic encephalopathy at presentation***				
Yes	141 (68.8%)	153(13.9%)	294 (22.5%)	
No	64 (31.2%)	946(86.1%)	1010(77.5%)	<0.01
***Presence of upper gastrointestinal bleed***				
Yes	37 (18.0%)	472(42.9%)	509 (39.0%)	
No	168 (82.0%)	627(57.1%)	795 (61.0%)	<0.01
***Tracheobronchial Aspiration during hospitalization***				
Yes	79 (38.5%)	15 (1.4%)	94 (7.2%)	<0.01
No	126 (61.5%)	1084(98.6%)	1210 (92.8%)	

* Chi-square test for independence was used.

A logistic regression was performed to ascertain the effect of gender, age, history of diabetes mellitus, etiology of cirrhosis, presence of hepatic encephalopathy at presentation, presence of upper GI bleed, and tracheobronchial aspiration on the likelihood that death would be the outcome of the hospitalization in liver cirrhosis patients. The logistic regression model was statistically significant, p<0.05. The model explained 38.8% (Nagelkerke *R^2^*) of the variance in group of patients who died and correctly classified 89.2% of cases. Patients who had tracheobronchial aspiration were 12.392 times more likely to die than who had no tracheobronchial aspiration. Similarly, patients who presented in hepatic encephalopathy were 7.862 times more likely to die than who presented without hepatic encephalopathy (Table-III).

**Table III:** Binary logistic regression output with Co-efficient, odds ratio and their 95% CI.

Risk Factors	B	S.E.	Wald-Statistic	p-value	Odds Ratio	95% C.I. for EXP(B)	

						Lower	Upper
Age	-0.006	0.007	0.901	0.343	0.994	0.980	1.007
Gender (Male/Female)	-0.266	0.196	1.828	0.176	0.767	0.522	1.127
Diabetes mellitus (Yes/No)	0.337	0.262	1.646	0.199	1.400	0.837	2.342
Etiology of cirrhosis (Viral/non-viral)	-0.166	0.256	0.419	0.517	0.847	0.513	1.399
Hepatic encephalopathy at presentation (Yes/No)	2.062	0.253	66.538	<0.01	7.862	4.790	12.904
Upper gastrointestinal bleed (Yes/No)	0.353	0.262	1.811	0.178	1.423	0.851	2.380
Tracheobronchial aspiration (Yes/No)	2.517	0.341	54.625	<0.01	12.392	6.357	24.157
Constant	-1.813	0.626	8.387	<0.01	0.1643		

Nagelkerke R Square = 22.6%, Cox & Snell R Square = 38.8%.

## DISCUSSION

Liver cirrhosis has a high inpatient mortality rate world-wide. In 2016, Cristal L. Brown and his colleagues^6^ from North Carolina, USA reported 13.5% inpatient mortality among cirrhotic patients. Similarly, in 2017, Zubieta-Rodriguez and colleagues^11^ from Colombia, and in 2011, Alsultan et al and colleagues^7^ from Riyadh, Saudi Arabia had demonstrated 23.5%, and 35% mortality among admitted cirrhotic patients respectively. In our study, we found 15.72% mortality rate among hospitalized cirrhotic patients. Multiple factors affect the outcome of hospitalization in these admitted cirrhotic patients. Alsultan MA et al observed worse outcome of hospitalization in cirrhotic patients who had worse CTP score, worse MELD score, and advanced age.^7^ They also found that advanced age (p=0.004) was an independent risk factor for the mortality of cirrhotic patients. Similarly, Chen CY and colleagues^12^ found that age > 75 years was significantly correlated with in-hospital mortality. In our study, mean age of the hospitalized cirrhotic patients who died was significantly higher than that of who not died (p<0.01). It seems that advanced age is always a risk factor for the mortality of cirrhotic patients world-wide.

When we applied logistic regression, only two factors (hepatic encephalopathy and tracheobronchial aspiration) were significantly predictive of death in liver cirrhosis patients. In 2017, Bajaj JA and colleagues^13^ found the hepatic encephalopathy as major determinant of mortality among cirrhotics. In our regression analysis of seven predictors, maximum odds ratio was 12.39 times higher death rate amongst patients who aspirated than who did not aspirated. We know that tracheobronchial aspiration is a preventable, but commonly occurring event in critically ill patients,^14^ and itself carries a 30% mortality risk.^15^ In hospitalized cirrhotic patients, this tracheobronchial aspiration is multifactorial; A big reason of tracheobronchial aspiration in comatosed cirrhotic patients is the large oral doses of lactulose administered to revert hepatic encephalopathy.^16^ The mode of administration of lactulose to revert hepatic encephalopathy should be individualized to prevent its aspiration. Secondly, tracheobronchial aspiration is observed during massive upper GI bleed which can be prevented by intubating^17^-^20^ patients if bleeding patients suddenly collapse. Some cirrhotic patients exhibit aspiration during endoscopic interventions^21^ which can also be minimized by adequate safety measures.^22^,^23^ Mortality from liver diseases is one of the leading cause of death in our country. A large sample study from Shifa International Hospital Islamabad showed that out of 8529 admissions; in-hospital mortality was reported in 283 (3.31%) patients. Most common cause was Out of these, 160 deaths were pertaining to medical causes. Out of these medical cases, 33 (20.6%) patients had died of chronic liver disease.^24^ Another study from same institute showed that there were 1294 (3.95%) mortalities. Out of which, 966 (74.65%) were due to various medical causes. Out of 966 deaths, 99 (10.24%) were due to Chronic Liver Disease.^25^

Our data suggested hepatic encephalopathy and tracheobronchial aspiration as independent predictors of mortality among hospitalized cirrhotic patients where hepatic encephalopathy is manageable and aspiration is a preventable condition. Hence, mortality rate among hospitalized cirrhotic patients can be reduced by adequate measures discussed above.

## CONCLUSION

The inpatient mortality rate amongst cirrhotic patients was high. Age, gender, history of diabetes, viral etiology of cirrhosis did not significantly contribute in the mortality of these patients. The patients who presented in hepatic encephalopathy and who suffered tracheobronchial aspiration during hospitalization were more likely to die. Excellence in hepatic encephalopathy management and prevention from aspiration can effectively reduce the mortality rate of cirrhotic patients in our hospitals.

### Authors’ Contribution

**AM and MAN:** conceived the idea and helped to design the study with MI and QR and review the manuscript.

**YM, RSMK and QR:** Data collection, write initial manuscript and review the final manuscript.

**YM and MI:** Performed statistical analysis of data and review the final manuscript.

**AM, MAN and MI:** Made the final editing of manuscript and review the final manuscript.

All the authors take full responsibility and are accountable for all aspects of the work ensuring questions related to the accuracy or integrity of any part of the work are appropriately investigated and resolved.
